# Supplementary material for: Content-rich biological network constructed by mining PubMed abstracts
Source: BMC Bioinformatics. 2004 Oct 8;5:147. doi: 10.1186/1471-2105-5-147 (PMC528731; doi:10.1186/1471-2105-5-147)
Supplement: Additional File 5 — The original Chilibot query results of the term "long-term potentiation (LTP)" and 22 other terms, limiting the latest references analyzed to the years 1990, 1995, 2000, and 2004. [file 1471-2105-5-147-S5.bz2 › chilibotAdditionalFile5/ltp2000/html/CAMKII_CAMKIV.html]

 


 **CAMKII** and **CAMKIV** 
  
Found 9 abstracts in PubMed,  **9 abstracts were retrieved and analyzed**.  


---

 Search Google  |
 PDF files only 
|  EDU domain only 

---

**Interactive relationship** (e.g. stimulation, inhibition, etc)

- The specific  **CaMKII**  inhibitor KN 62 50 microM had no effect on the  **CaMKIV**  activity and pancreatic enzyme secretion elicited by CCK 8, CCh and CCK OPE.  Ref: 10838169 Biochim Biophys Acta, 2000
- The results indicate that  **CaMKIV**  is much more potent than  **CaMKII**  in activating CREB in three different cell lines.  Ref: 7958915 Genes Dev, 1994
- Because both  **CaMKII**  and  **CaMKIV**  can phosphorylate CREB, we pursued further the mechanism by which  **CaMKII**  and  **CaMKIV**  differentially regulate CREB activity.  Ref: 7958915 Genes Dev, 1994
- Similar anti proliferative effects of KN 62 occurred in SK N SH human neuroblastoma cells, which express both  **CaMKII**  and  **CaMKIV** , and in K562 human chronic myelogenous leukemia cells, which express  **CaMKII**  but not  **CaMKIV** .  Ref: 8615909 Biochem Pharmacol, 1996

**Parallel relationship** (e.g. studied together, co-existance, homology, etc.)

- Transfection experiments using expression plasmids for constitutively active forms of CaMKI,  **CaMKII** , or  **CaMKIV**  show that  **CaMKII**  is the most effective activator of prolactin promoter expression.  Ref: 9324052 Mol Cell Endocrinol, 1997
- The roles of two multifunctional calcium calmodulin dependent protein kinases,  **CaMKIV**  and  **CaMKII** , were examined in transient transfection studies that utilized either the full length or the constitutively active forms of these kinases.  Ref: 7958915 Genes Dev, 1994
- Immunoprecipitation and immunoblotting studies using a  **CaMKII**  or  **CaMKIV**  antibody showed that rat pancreatic acini expressed both  **CaMKII**  and  **CaMKIV** .  Ref: 10838169 Biochim Biophys Acta, 2000
- Calcineurin,  **CaMKII** , and  **CaMKIV**  activities increased at 2 minutes and peaked by 1.  Ref: 11073891 Circ Res, 2000
- When Ser142 was mutated to alanine, CREB was activated by  **CaMKII** , as well as by  **CaMKIV** .  Ref: 7958915 Genes Dev, 1994
- These findings indicated that LIF activated  **CaMKII** ,  **CaMKIV** , and calcineurin through an increase in I Ca, L and calcium i.  Ref: 11073891 Circ Res, 2000
- The expression of PKAalpha, PKCalpha and  **CaMKII** ... in periglomerular cells suggests that these three kinases may play a role in TH gene regulation in the olfactory bulb.  Ref: 10944503 Chem Senses, 2000
- Whereas  **CaMKIV**  was primarily found in granule cells,  **CaMKII**  was present in the glomerular, external plexiform, mitral cell and granule cell layers.  Ref: 10944503 Chem Senses, 2000
- The fairly high degree of the substrate specificity of this protein phosphatase suggests that the physiological significance of the phosphatase may be the regulation of the three multifunctional calcium calmodulin dependent protein kinases, CaMKI,  **CaMKII** , and  **CaMKIV** , which are the key enzymes in a calcium signaling system in the cell.  Ref: 9875237 Biochem Biophys Res Commun, 1998
- The expression of both  **CaMKII**  and  **CaMKIV**  by SCLC cells, and the sensitivity of these cells to the anti proliferative effects of KN 62, suggest a role for CaM kinase in regulating SCLC proliferation.  Ref: 8615909 Biochem Pharmacol, 1996
- Examination of the subcellular distribution of transiently expressed  **CaMKIV**  and  **CaMKII**  reveals that only  **CaMKIV**  enters the nucleus.  Ref: 8065343 Mol Cell Biol, 1994
- We also demonstrated that SCLC cells express both CaM kinase type II  **CaMKII**  and CaM kinase type IV  **CaMKIV** .  Ref: 8615909 Biochem Pharmacol, 1996
- While  **CaMKII**  has been implicated in learning and memory, the biological role of the other multifunctional CaM kinases, CaMKI and  **CaMKIV** , is largely unknown.  Ref: 10673339 Genomics, 2000
- Although  **CaMKII**  and  **CaMKIV**  use autocamtide II as a substrate, peptide gamma is more efficiently phosphorylated by  **CaMKIV**  than by  **CaMKII** .  Ref: 10838169 Biochim Biophys Acta, 2000
- **CaMKII** ,  **CaMKIV** , and calcineurin are critically involved in LIF induced cardiac hypertrophy.  Ref: 11073891 Circ Res, 2000
- KN62 an inhibitor of  **CaMKII**  and  **CaMKIV**  attenuated c fos, brain natriuretic peptide, alpha skeletal actin, and atrial natriuretic peptide expression.  Ref: 11073891 Circ Res, 2000
- The aim of this study was to investigate whether LIF activates calcium dependent signaling molecules, such as calcineurin and calmodulin kinases II and IV  **CaMKII**  and  **CaMKIV** , and, if so, whether these calcium mediated signaling events contribute to LIF mediated cardiac hypertrophy.  Ref: 11073891 Circ Res, 2000
